# Are food exposures obtained through commercial market panels representative of the general population? Implications for outbreak investigations

**DOI:** 10.1017/S0950268819000219

**Published:** 2019-02-22

**Authors:** T. Inns, D. Curtis, P. Crook, R. Vivancos, D. Gardiner, N. McCarthy, P. Mook

**Affiliations:** 1 Field Service, Public Health England, London, UK; 2 NIHR Health Protection Research Unit in Gastrointestinal Infections, University of Liverpool, Liverpool, UK; 3 Institute of Psychology, Health and Society, University of Liverpool, Liverpool, UK; 4 NIHR Health Protection Research Unit in Emerging and Zoonotic Infections, University of Liverpool, Liverpool, UK; 5 Warwick Medical School, University of Warwick, Coventry, UK

**Keywords:** Case-control studies, epidemiological study design, gastrointestinal infection, outbreaks

## Abstract

Current methods of control recruitment for case-control studies can be slow (a particular issue for outbreak investigations), resource-intensive and subject to a range of biases. Commercial market panels are a potential source of rapidly recruited controls. Our study evaluated food exposure data from these panel controls, compared with an established reference dataset. Market panel data were collected from two companies using retrospective internet-based surveys; these were compared with reference data from the National Diet and Nutrition Survey (NDNS). We used logistic regression to calculate adjusted odds ratios to compare exposure to each of the 71 food items between the market panel and NDNS participants. We compared 2103 panel controls with 2696 reference participants. Adjusted for socio-demographic factors, exposure to 90% of foods was statistically different between both panels and the reference data. However, these differences were likely to be of limited practical importance for 89% of Panel A foods and 79% of Panel B foods. Market panel food exposures were comparable with reference data for common food exposures but more likely to be different for uncommon exposures. This approach should be considered for outbreak investigation, in conjunction with other considerations such as population at risk, timeliness of response and study resources.

## Introduction

Outbreaks of infectious disease require timely investigation and rapid implementation of control measures to protect public health. For foodborne outbreaks of infectious gastrointestinal disease, there is a need for comparison food exposure data. The controls recruited to obtain this information must be representative of the population at risk of the disease [1]. In countries such as Australia and The Netherlands, nationally representative population databases and surveys are routinely used to support infectious disease surveillance [2, 3]. By contrast, current methods of control recruitment in England include case-nominated controls [4], systematic digit dialling [5] and convenience sampling [6]. In addition to being resource-intensive and subject to potential biases [7], these methods are often slow. Given that the speed of investigation and identification of the source can be vital for stopping severe outbreaks [8], the increasing use of internet-based methods to recruit controls may provide a way of obtaining vital information in a more timely fashion.

Internet-based questionnaires offer numerous potential advantages over paper questionnaires for the collection of epidemiological data [9]. Given these advantages, internet-based questionnaires may be a suitable alternative to some current epidemiological data collection methods [10]. One method of obtaining internet-based questionnaire data is via commercial market research panels. These panels comprise individuals who have elected to receive online questionnaires and can opt to complete a given questionnaire in return for a reward. Although most often used by marketing companies or polling organisations to obtain information about a target audience, these panels have also been used to collect epidemiological exposure data. This was first reported in 2010 for a retrospective case-control study of listeriosis in England [11] and first reported during an outbreak investigation in 2012 [12]. Market panels have been used as a rapid method of control recruitment when investigating gastrointestinal outbreaks in the UK [12–15] and Japan [16].

Although the logistical issues associated with using these controls have been reviewed [17], there has thus far been no systematic assessment of the quality of food exposure information obtained from these market panel recruited controls compared with a gold standard. Without this information there is a question as to whether these controls are comparable with the general population and thus representative of the population at risk. This is important, as a poor choice of controls can lead to both incorrect results and possible medical harm [18]. The aim of this study was to evaluate the usefulness of market panel-recruited controls by comparing the distribution of food exposures collected from these panels with an established dataset considered to be representative of the eating habits of people in England.

## Methods

### Data collection

Market panel data were collected using internet-based surveys administered by two different international market research companies. Methods of data collection have been described previously [12]. Briefly, both companies (‘A’ and ‘B’) have panels of respondents who are registered with the company and targeted subsets of this population are sent links to online questionnaires. Market panel respondents who complete the questionnaire are rewarded for their time. The market panel surveys were compared with reference data from the National Diet and Nutrition Survey (NDNS), a survey conducted to measure the diet and nutritional intake of people in the UK.

The NDNS methods are described fully elsewhere [19]. Briefly, NDNS data were collected on a rolling annual basis from 2008 to 2014 [20]. Each year a new cohort was randomly selected from the population and asked to complete a food and drink diary at various points throughout the year. Participants were interviewed face-to-face and then asked to prospectively record types and quantities of foods consumed in a 4-day paper-based food diary.

Data from NDNS are publically available upon registration. Individual-level, anonymised data were downloaded from the data repository [20]. We restricted data from the NDNS to only those persons aged over 17 and resident in England. Age, sex and Index of Multiple Deprivation (IMD) quintile were included. Individual food exposure data were recoded to binary exposure status (yes/no) for 71 selected foods.

Sample sizes for market panel surveys A and B were calculated to detect an odds ratio (OR) of 1.5 with power of 0.8 and *α* of 0.05, based on the proportion of the reference population exposed to foods commonly associated with outbreaks of foodborne illness. The required sample sizes of market panel respondents were stratified to reflect the geographical and sex distribution of the population of England.

For both market panel surveys, data collection took place over three time periods to capture seasonal differences (so as to make comparison with NDNS); August 2017, December 2017 and March 2018. In each of these months, respondents from both companies were recruited over 4-day periods. In the online questionnaire, participants were asked for their age, sex and postcode; this postcode was used to infer IMD quintile of residence. Participants were asked whether they had consumed each of 71 selected foods over the previous 4 days. Responses were restricted to those aged over 17 years and resident in England, to match criteria applied to the NDNS data. The online questionnaire took approximately 10 min to complete.

### Analysis

Study participants were described in terms of age, sex and IMD quintile of residence. Statistically significant differences, at the 5% level, in the distribution of these variables between the market panel and NDNS participants were tested using a *t* test (age), a *χ*^2^ test (sex) and a *χ*^2^ test for trend (IMD quintile). The proportion exposed to each of the 71 foods was described for both reference and market panel datasets. We used ORs to compare exposure to each food item between the market panel and NDNS participants. Using logistic regression, adjusted ORs (aORs) with 95% Confidence Intervals were calculated for each of the 71 foods, with the association between food exposure and dataset simultaneously adjusted for age, sex and IMD quintile. The 71 foods were grouped into seven food groups; carbohydrate, dairy and egg, seafood, fruit, meat, vegetables and other. We used a fixed-effects logistic regression model to produce pooled estimates of association for each of these seven food groups. If the 95% Confidence Interval of the aOR did not include 1, we inferred that this was the evidence of statistical significance. Given the large ORs typical of exposures leading to intestinal infectious disease outbreaks [5, 8, 21], we defined that ORs within the range from 0.3 to 3.0 were of limited practical importance; if the 95% Confidence Interval of the aOR did not include this range, we used this to infer evidence of practical importance.

For each market panel survey, the difference in exposure percentage between the panel and NDNS participants was compared against the average exposure percentage of the panel and NDNS participants using a Bland-Altman plot [22]. The mean exposure percentage difference for all 71 foods in each panel was compared with 0 using a two-tailed *t* test. The assumption that the difference in exposure percentage is the same across the range of exposure percentages was tested using linear regression. All analyses were carried out in R 3.4.1 [23]; the *metafor* [24] package was used for calculating pooled ORs.

### Ethical considerations

This study is an evaluation of a service that has been used for public health practice. It does not involve patients, treatments, samples, investigations or any element of care; this study does not involve any element of randomisation. As such, the study did not require Research Ethics Committee review.

## Results

### Participant demographics

Overall, the analysis included NDNS food diary data for 2696 English residents. For both market research panels, the number of responses exceeded the per-panel target of 2103 and met the targets for each sex and geographic region stratum.

The demographic characteristics of participants are presented in Table 1. Panel A participants were significantly older (mean age 54.1 years) than NDNS participants (mean age 48.1); there was no significant difference in mean age between NDNS and Panel B participants. Both Panels A (49%) and B (48.4%) had a significantly higher proportion of male participants than did NDNS (42.5%). IMD quintile 1 indicates the most deprived area; 21.8% of NDNS participants were from this quintile, compared with 17.6% of Panel A participants and 24.7% of Panel B participants. Panel B participants were significantly more likely to be from more deprived areas (*P* < 0.001) than NDNS participants, whereas Panel A participants were significantly more likely to be from less deprived areas (*P* = 0.003).

**Table 1.** Description of demographic characteristics in NDNS, Panel A and Panel B participants

	Level	NDNS	Panel A	*P*-value	Panel B	*P*-value
*n*		2696	2157		2274	
Age mean (s.d.)		48.1 (17.99)	54.1 (42.86)	<0.001	47.21 (82.83)	0.598
Sex *n* (%)	Female	1549 (57.5)	1099 (51.0)	<0.001	1174 (51.6)	<0.001
	Male	1147 (42.5)	1058 (49.0)		1100 (48.4)	
IMD quintile *n* (%)	1	588 (21.8)	362 (17.6)	0.003	535 (24.7)	<0.001
	2	551 (20.4)	414 (20.1)		536 (24.7)	
	3	493 (18.3)	407 (19.8)		412 (19.0)	
	4	511 (19.0)	443 (21.6)		372 (17.2)	
	5	553 (20.5)	429 (20.9)		312 (14.4)	

### Food exposures

Of the total 71 foods included in the study, the median number of items consumed per participant was comparable for Panel A (27, interquartile range (IQR) 21–34) and Panel B (25, IQR 20–31). Both were higher than reported for NDNS (20, IQR 16–24).

**Fig. 1.**
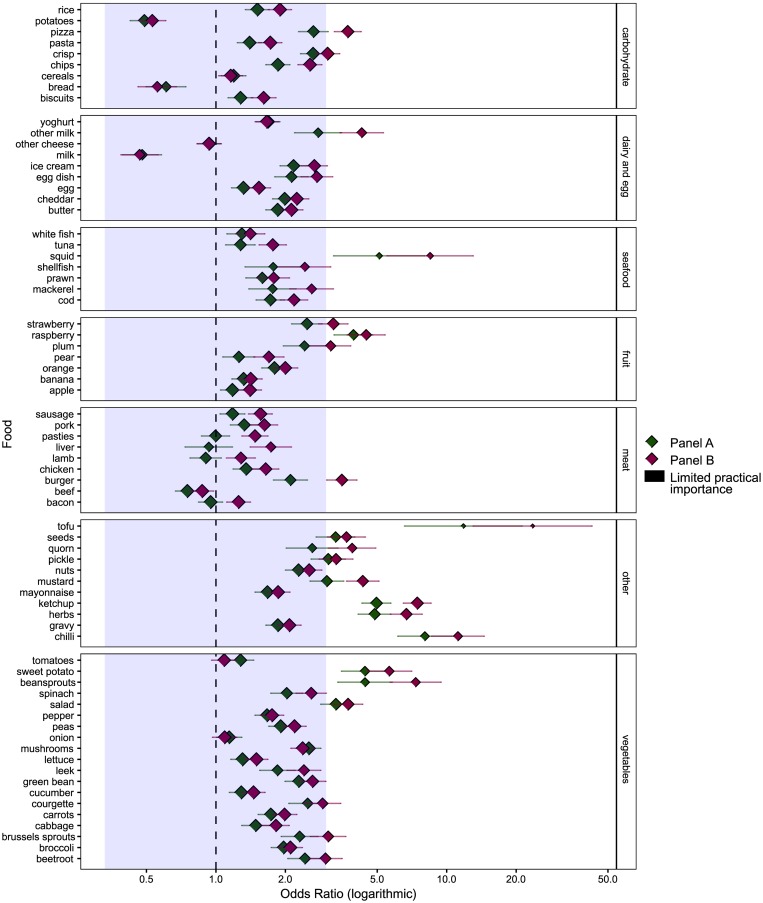
Adjusted ORs of individual food exposures between the market panel and NDNS participants.

The aORs for the association between each of the 71 food exposures and dataset are shown in Figure 1, with a full table of results in Supplementary Table 1. The majority of food items (90%) were more often reported as eaten by panel participants than NDNS participants; 87% of foods for Panel A and 93% of foods for Panel B. The highest aORs were reported for tofu (Panel A aOR 11.81 (95% CI 6.57–21.25); Panel B aOR 23.57 (13.02–42.69)), chilli (Panel A aOR 8.05 (6.14–10.54); Panel B aOR 11.19 (8.60–14.55)) and squid (Panel A aOR 5.1 (3.24–8.04); Panel B aOR 8.47 (5.51–13.03)). These were also the least consumed items by NDNS participants.

Only four items (6%) were reported as consumed less frequently by panel participants than NDNS participants: milk (Panel A aOR 0.48 (95% CI 0.39–0.58); Panel B aOR 0.47 (0.39–0.56)), potatoes (Panel A aOR 0.49 (0.43–0.56); Panel B aOR 0.53 (0.46–0.61)), bread (Panel A aOR 0.61 (0.50–0.74); Panel B aOR 0.56 (0.46–0.67)) and beef (Panel A aOR 0.75 (0.67–0.85); Panel B aOR 0.87 (0.78–0.98)). Three of these foods (bread, milk and potatoes) were the most commonly consumed items in the study. For seven items (10%) there was no significant difference in exposure odds between the market panel and NDNS participants. There was no evidence of differences of practical importance for 63/71 (89%) of foods for Panel A and 56/71 (79%) of foods for Panel B using our criteria of a threefold difference in odds of reported consumption. Eight foods were substantially different in both panels (Squid, Beansprouts, Sweet potato, Raspberry, Tofu, Herbs, Ketchup, Chilli).

For both Panels A and B there was no significant difference in reported exposure odds for ‘other’ (non-cheddar) cheese (Panel A aOR 0.94 (95% CI 0.83–1.06)), as compared with NDNS. Among Panel A participants there was no evidence of a difference in odds of several meat products: pasties (aOR 1.00 (95% CI 0.87–1.15)), liver (aOR 0.93 (95% CI 0.74–1.18)), lamb (aOR 0.90 (95% CI 0.77–1.06)) and bacon (aOR 0.95 (95% CI 0.84–1.07)). For Panel B participants the odds of exposure to tomatoes (aOR 1.09 (95% CI 0.96–1.23)) and onion (aOR 1.09 (95% CI 0.97–1.23)) were not significantly different from those in NDNS participants.

**Fig. 2.**
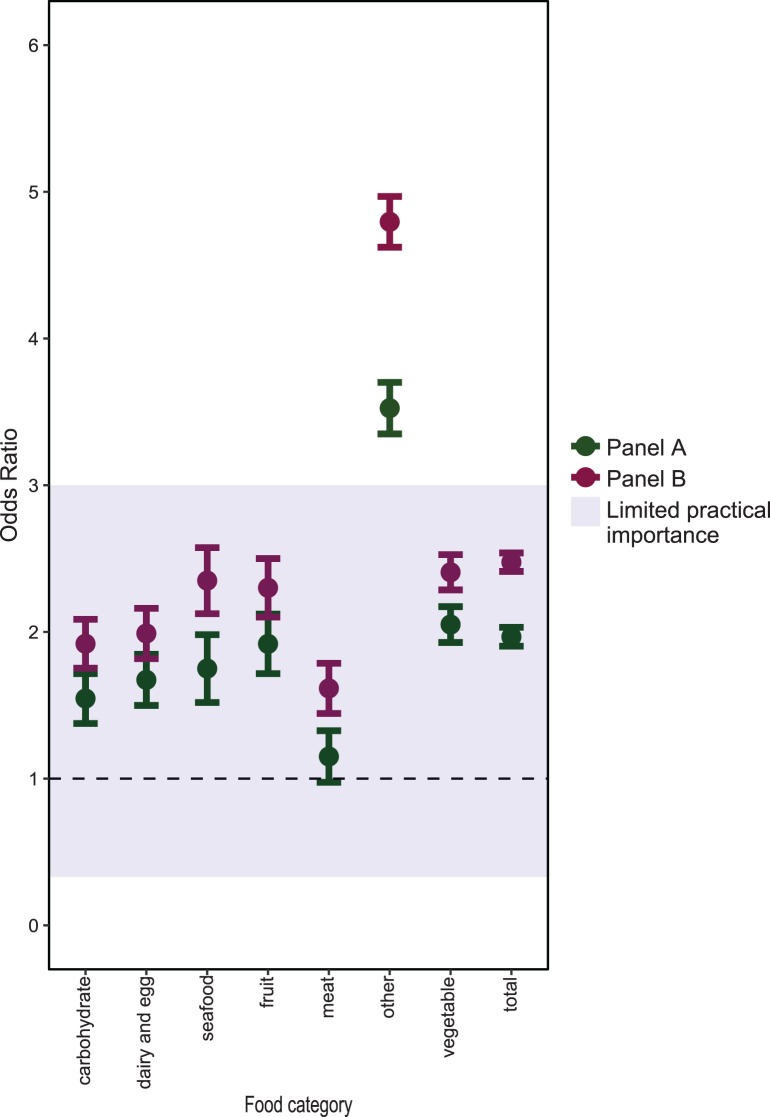
Adjusted ORs of food exposures, pooled by food category, between the market panel and NDNS participants.

In the pooled analysis (Fig. 2), both Panel A and B participants had more than twice the odds of reported exposure to items classified as vegetables (Panel A aOR 2.05 (95% CI 1.93–2.17); Panel B aOR 2.41 (2.29–2.53)), compared with NDNS participants. Among Panel A participants, there was no evidence of a difference in the pooled aOR for items classified as meat products (OR 1.15 (95% CI 0.98–1.33)). For all seven food categories, the pooled aORs for Panel B were statistically significantly different from 1. However, for all categories apart from ‘other’, there was limited evidence that these differences were practically important.

**Fig. 3.**
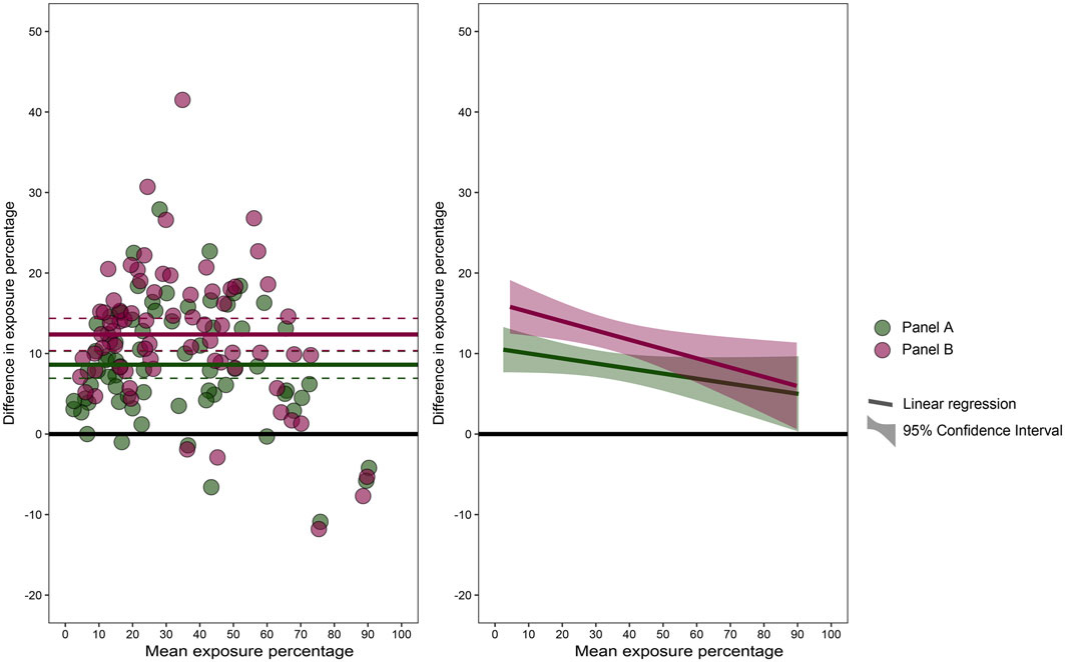
Bland-Altman plot shows mean exposure percentage of panel and NDNS participants against the difference in exposure percentage of panel and NDNS participants. (i) Shows the difference in mean percentage exposure for each panel. (ii) Shows a linear regression testing the relationship between the difference in exposure percentage and the mean exposure percentage for each panel.

For both market panel surveys, the difference in exposure percentage between the panel and NDNS participants was compared against the average exposure percentage in a Bland-Altman plot (Fig. 3). The mean exposure percentage difference (Fig. 3i) was significantly different from 0 for both Panel A (8.61, *P* < 0.001) and Panel B (12.37, *P* < 0.001). The results of linear regression to test the assumption that the difference in exposure percentage is the same for uncommon and very common foods are shown in Figure 3ii. For Panel A, there was no evidence that the difference in exposure percentage was different for more or less frequently reported foods (coefficient = 0.54, *P* = 0.103, adjusted *R*^2^ = 0.024). Among Panel B participants, there was some statistical evidence that more frequently reported foods had a smaller difference in exposure percentage (coefficient = 0.48, *P* = 0.014, *R*^2^ = 0.072).

## Discussion

This paper presents the results of a novel comparison between food exposure data from market panel recruited controls and an established population survey. This is an important comparison given the increasing use of these approaches in outbreak investigation in the UK and internationally [17]. We found that when adjusted for socio-demographic factors, there were statistically significant differences in the percentage of participants exposed to 90% of foods between both panels and the reference data; however, in the context of large ORs typically associated with foodborne exposures leading to outbreaks the difference was practically important (a greater than threefold difference in odds of reported exposure) for only 11% of foods. Although the two panels gave largely similar results, responses from Panel A participants mirrored more closely the reference data than responses from Panel B participants.

We found that foods that were reported much more frequently by market panel participants (reporting their consumption over 4 days retrospectively) than the reference data included less commonly consumed foods such as tofu, chili, squid and beansprouts. We also found that commonly consumed foods such as bread and milk were reported more frequently by NDNS participants (from their prospective food diaries) than market panel participants. Looking at categories of food, we found that the panel data were most similar to the reference data for meat, carbohydrates and dairy/egg products. Of the 71 individual foods included in the study, those for which the market panel participants were most similar to the reference data included onion, tomatoes, bacon, beef, lamb, liver, other cheese and cereals. Based on recent foodborne outbreaks in England [25], the most common food vehicles associated with gastrointestinal outbreaks are poultry meat, red meat and crustacea/shellfish. For these foods we found that the differences between panel control and NDNS were not of practical significance, so would support using these control recruitment methods for outbreaks where these were a hypothesised vehicle.

In addition to statistical significance, we also defined ORs within the range from 0.3 to 3.0 as of limited practical significance. The rationale for this was that although differences between groups may have been statistically significant, often in foodborne outbreak investigations the associations will have very large ORs and therefore be comparatively unaffected by very small but statistically significant differences in exposure proportions. However, there may be some foodborne outbreaks with smaller ORs which are diluted by misclassification of cases and the arbitrary nature of this range of practical significance should be interpreted in this light. Given that food exposures were reported more frequently in panel control than by NDNS participants it is possible that using market panel controls during a foodborne outbreak investigation could lead to an underestimation of the association between food and illness.

When interpreting these results it is important to consider the possible biases that may have affected this study. The main differences in panel data and the reference data collection were: the use of an internet tool *vs.* use of a paper-based tool and the retrospective *vs.* prospective nature of data collection. Differences in the resulting food exposure results may be due to a combination of these factors and it is difficult to isolate their separate effects. Although previous work has demonstrated the equivalence of self-reported paper and internet data collection methods, particularly in psychology studies [26], this is a possible source of information bias. In this study, the food exposure data from market panel participants was collected and coded in a different way to that from NDNS participants. NDNS data participants had training in completing a food diary; information entered into this diary was then coded and classified by trained study staff [19]. Market panel participants received no training and completed questions directly asked about the consumption of 71 foods. Although all food items would be affected, it may also be that those NDNS participants with personal contact could under-report ‘bad’ food items and over-report ‘good’ food items if they experienced a social desirability response bias.

Collecting the data retrospectively for the panel controls may have introduced recall bias, if participants included foods they ate relatively recently, rather than strictly those foods consumed in the previous 4 days, leading to overestimation of the number of items eaten. Indeed there was evidence that panel controls recalled more items. It is possible that on this basis, unusual or exotic foods may be over-remembered compared with common food items. If this is a factor which explains the significant differences in our study then such overestimation may not be a concern in outbreak studies where data are similarly collected retrospectively.

If there are differences in the results due to inherent sampling biases then this could compromise using panel controls in case-control studies. This study highlights that despite adjusting for age, sex and a proxy for socio-demographic status, there was variation by individual food items that is hard to explain due to the biases described above e.g. tofu and vegetables in general compared with meat. Such dietary choices may have been affected by cultural background; in this study it was not possible to collect this information from panel control and it was therefore not possible to adjust for it in this analysis. Other studies report selection biases which stem from the way in which individuals who volunteer to join these panels systematically differ from those that do in a variety of ways, including food exposures [27, 28]. If market panel controls are used, this should be considered as this cannot be controlled for via study design. When interpreting the results from outbreak studies using panel controls, it is therefore important to consider whether the measure of effect for particular food items may have been due to such inherent sampling issues, making use of our study results and other similar studies.

An additional limitation of this study is the possible confounding due to changes in diet over time. The most recent NDNS data available at the time of this study was from 2008 to 2014. The market panel participants were recruited between August 2017 and March 2018. Changes in dietary trends over this period could at least partially account for the large effect sizes of items such as tofu and beansprouts, but although there has been an anecdotal increase in vegetarianism, only 2.6% of adults in the UK were vegetarian in 2014 [20] and no recent reliable estimates are available.

This study did not aim to formally compare food exposures of the two panels against each other. There are multiple international market research panel companies from which participants could have been selected, all with different recruitment and sampling methodologies; the two panels we used may not be representative of all panels, although the largely similar results across the two panels suggest that these results may at least in part represent a general estimate for internet panel data in this area. There are minor apparent differences between the two panels used in this study for some exposures and this may be a result of differing recruitment and exclusion policies. Multiple market panel companies provide a similar service and the choice for which, if any, panel to use in an outbreak situation should be based on considerations of cost, timeliness and feasibility, in addition to the findings presented here.

Issues previously identified with using market research panels include panel responsiveness, socio-demographic representativeness, careless responses, overlap between panel membership and biases due to recruitment [29, 30], though market research companies attempt to address these issues through routine practice [17]. One of the key benefits of this approach, however, is the efficiency with which large numbers of controls can be rapidly recruited with little public health resource [12]. It can also identify associations that can be further validated by evidence from parallel epidemiological, trace-back or microbiological studies [13].

We identified – and quantified – differences between the food exposures of panel controls and the NDNS. Similar, well-documented differences between exposures of traditionally-recruited controls and the general population also exist, however, these biases are generally better understood [7]. As such, it is important that further work be done to quantify differences between exposures of controls recruited through market research panels and traditional methods and biases in obtaining case data which may also vary according to the method of collection. Prior quantification of differences between controls recruited through market research panels and controls recruited from Public Health England staff indicated that there were differences in only 29% of the compared exposures but, as the bias associated with the latter control group was not well understood, no conclusion could be made on biases associated with market research panel controls. Ideally one would be confident that if using market panel controls these are no more biased than current best practice and benefit from increased speed of recruitment/public health action with limited resource costs. It is also important to understand any likely bias introduced when using panel controls so that one could control for this in the design and analyses of such studies [17] or interpretation of results. This study is the first to evaluate likely bias resulting from the retrospective internet panel method and can offer data to support the interpretation of studies that use this approach to obtain control data. To isolate and better quantify this bias, future studies should compare panel and population data collected via similar means (e.g. both retrospective, electronic).

In conclusion, when adjusted for socio-demographic factors, retrospective food information gathered from market panels is comparable with prospective diary information from the general population for common food exposures, relative to the effect sizes expected from foodborne outbreak investigations. This is less true for uncommon exposures. Other considerations such as the context of the outbreak, required timeliness of the response and study resources should be factored in when considering using this approach for an outbreak investigation. Due to the limitations of this study, further work is required to validate the food exposures reported by market panels against data collected in a similar retrospective fashion from controls in outbreak investigation contexts. We will make the market panel data used in this study available as a tool for other epidemiologists to use as both a reference group for rapid case-control studies with food exposures as hypothesised sources and to make comparisons with other control food exposure information to this market panel data.

## Supplementary material

For supplementary material accompanying this paper visit https://doi.org/10.1017/S0950268819000219. click here to view supplementary material
